# 

**Published:** 1887-11

**Authors:** 


### CONTENTS:

ARTICLE I.—Capping Exposed Pulps. By George H. Cushing........ 289
" II.—Pulpless Teeth and their Treatment. By George H. Cushing ............ 294
" III.—Vacuum Plates. By W. E. Swigert, D. D. S ............ 299
" IV.—The Six Year Molars. By A. J. Smith, D. D. S.... ...... 302
" V.—Antisepsis. By M. H. Chappell, D. D. S.......... ......... 306
" VI.—Operative Dentistry as Applied to Deciduous Teeth. By Dr. Wm. N. Morrison ........................ 312
" VII.—The Use of the Microscope in Progressive Dentistry. By L. L. Davis, D. D. S...... ..... 316
" VIII.—A Visit to Foreign Dental Schools and other Observations. By A. W. Harlan, M. D., D. D. S. 320
" IX.—Are There Too Many Dentists? .... ...... 324
" X.—An Artificial Crown. By A. W. McCandless, D. D. S... 326
" XI.—Cases in Practice. By I. P. Wilson........... 328
The First District Dental Society of the State of New York..... ........ 331

### EDITORIAL, ETC.

Dental Department of the University of Maryland....... ..... 332
Indirect and Improper Advertising.......... 332

### OBITUARY.

D. M. Parker, D. D. S...... . 333

### MONTHLY SUMMARY.

Trophic Nerves ........ 334

Death During Ether Anæsthesia........ 335

Death of a Lady After Having Seventeen Teeth Extracted........ 336

Effects of Study on the Teeth ........ 336

A Dentist's Bill........ 336

